# Salvage pleurectomy/decortication following immunotherapy for malignant pleural mesothelioma

**DOI:** 10.1093/icvts/ivad173

**Published:** 2023-11-15

**Authors:** Masaru Takenaka, Koji Kuroda, Katsuma Yoshimatsu, Masataka Mori, Masatoshi Kanayama, Akihiro Taira, Taiji Kuwata, Fumihiro Tanaka

**Affiliations:** Second Department of Surgery (Chest Surgery), University of Occupational and Environmental Health, Kitakyushu, Japan; Second Department of Surgery (Chest Surgery), University of Occupational and Environmental Health, Kitakyushu, Japan; Second Department of Surgery (Chest Surgery), University of Occupational and Environmental Health, Kitakyushu, Japan; Second Department of Surgery (Chest Surgery), University of Occupational and Environmental Health, Kitakyushu, Japan; Second Department of Surgery (Chest Surgery), University of Occupational and Environmental Health, Kitakyushu, Japan; Second Department of Surgery (Chest Surgery), University of Occupational and Environmental Health, Kitakyushu, Japan; Second Department of Surgery (Chest Surgery), University of Occupational and Environmental Health, Kitakyushu, Japan; Second Department of Surgery (Chest Surgery), University of Occupational and Environmental Health, Kitakyushu, Japan

**Keywords:** Salvage surgery, Pleurecotomy/decortication, Mesothelioma, Immunotherapy

## Abstract

Salvage surgery following immunotherapy is a promising treatment option for advanced malignant tumour. However, only a few cases of salvage surgery for malignant pleural mesothelioma (MPM) have been reported. This retrospective study was conducted to assess the feasibility of salvage surgery following immunotherapy for initially unresectabele MPM. Among 61 patients who received pleurectomy/decortication (P/D) for MPM, 7 patients received salvage P/D after immunotherapy. Surgical indication of salvage P/D was conversion to resectability in 5 patients and local relapse in 2 patients, and macroscopic complete resection was achieved in all patients. Although salvage P/D was associated with longer operation time (median, 507 min), higher intraoperative blood loss (median, 2573 mL) and higher morbidity (≥ grade 3, 29%), no patient died after surgery. Radiographic response to immunotherapy was well correlated with pathologic response, as all 4 patients with partial response showed significant pathologic response (viable cells, ≤50%). With the median postoperative follow-up duration of 9.0 months, all patients were alive mostly without tumour recurrence as local recurrence developed in 1 patient. To conclude, salvage P/D after immunotherapy may be a feasible treatment option for selected patients with advanced MPM, which should be validated in future multi-institutional studies. In addition, a long-term follow-up is essential to reveal the clinical benefit achieved with salvage P/D following immunotherapy.

## INTRODUCTION

Malignant pleural mesothelioma (MPM) is a highly aggressive malignant tumour associated with exposure to asbestos. Curative-intent surgery, extrapleural pneumonectomy (EPP) or pleurectomy/decortication (P/D) may be offered for highly selected patients. For the majority of patients, systemic treatment may be indicated, but standard platinum-based chemotherapy provides only a modest clinical benefit [1]. Recently, a pivotal study demonstrated that first-line immunotherapy using immune checkpoint inhibitors, nivolumab plus ipilimumab, provided a superior overall survival benefit over standard chemotherapy [2]. In addition, for patients who progressed after chemotherapy, nivolumab monotherapy provided a significant survival benefit [3].

Salvage surgery after initial treatment may provide some clinical benefit for selected patients with initially unresectable malignant disease. In fact, the feasibility and efficacy of salvage surgery for advanced non-small-cell lung cancer following effective systemic treatment (targeting therapy for mutation-positive patients [4] or immunotherapy for mutation-negative patients [5]) have been reported. However, only a few case reports of salvage surgery after immunotherapy in MPM have been reported; Banks *et al.* [6] reported a case of a salvage EPP after immunotherapy (nivolumab plus ipilimumab), and we reported a case of salvage P/D after the same immunotherapy [7]. For resectable MPM, P/D has been preferably employed due to its lower morbidity and mortality [1]. P/D may be more preferable in a salvage setting after immunotherapy, because immune-related adverse events such as pneumonitis can be fatal if they develop after EPP. This study was conducted to assess the feasibility of salvage P/D after immunotherapy for initially unresectable MPM.

## MATERIALS AND METHODS

We retrospectively analysed patients who underwent curative-intent surgery for MPM between January 2017 and March 2023 at our institute (Supplementary Material, Methods). Salvage P/D was indicated, if each patient could tolerate P/D and achievement of macroscopic complete resection (MCR) with P/D was expected by preoperative assessment. Patients who underwent salvage surgery met the following criteria: (i) prior systemic treatment with any immune checkpoint inhibitor; (ii) no a priori plan to perform curative-intent surgery; and (iii) confirmation of loco-regional recurrence or persistent tumour and no other extra-thoracic lesion [4]. This study was approved by the institutional review board of our institute (UOEHCRB19-042).

## RESULTS

Among a total of 61 patients who underwent curative-intent surgery (P/D in all patients), 7 patients received salvage P/D after immunotherapy (nivolumab plus ipilimumab in 5 patients and nivolumab following chemotherapy in 2 patients) (Supplementary Material, Table S1). The other 54 patients received P/D for initially resectable MPM (non-salvage P/D) (Table 1). Non-incisional P/D technique was employed to achieve *en bloc* resection of the entire pleura [8]. The resected diaphragm and pericardium were reconstructed, when extended P/D was performed to achieve MCR.

**Table 1:** Characteristics of patients who received pleurectomy/decortication

	Salvage P/D	Non-salvage P/D	*P*-Value
No. of patients	7	54	
Age (years), median (range)	64.0 (47–77)	68.0 (47–79)	0.379
Sex, *n* (%)			
Male	7 (100)	50 (92.6)	1.000
Female	0	4 (7.4)	
Histology, *n* (%)			
Epithelioid	6 (85.7)	40 (74.1)	0.670
Biphasic	0	4 (7.4)	
Sarcomatoid	1 (14.3)	10 (18.5)	
Performance status, *n* (%)			
0	1 (14.3)	23 (42.6)	0.229
1	6 (85.7)	28 (51.9)	
2	0	3 (5.6)	
Clinical stage, *n* (%)			
I	1 (14.3)	44 (81.5)	<0.001
II	0	8 (14.8)	
III	6 (85.7%)	2 (3.7)	
Systemic treatment prior to surgery, *n* (%)			
Yes	7 (100)	7 (14.9)	<0.001
Cis/PEM	0	7 (14.9)	
Nivo/Ipi	5 (71.4)	0	
Cis/PEM → Nivo	2 (28.6)	0	
Operation time (min), median (range)	507 (290–857)	375 (204–556)	0.030
Blood loss (ml), median (range)	2573 (420–5900)	1804 (490–4210)	0.269
Achievement of MCR with P/D, *n* (%)	7 (100)	47 (87.0)	0.856
Combined resection, *n* (%)			
Yes	7 (100)	51 (94.4)	1.000
Diaphragm	6 (85.7)	48 (88/9)	
Pericardium	5 (71.4)	33 (61.1)	
Azygous vein	2 (28.6)	1 (1.9)	
Rib	2 (28.6)	1 (1.9)	
Esophagus	0	1 (1.9)	
Pathologic stage, *n* (%)			
0	1 (14.3)	0	0.430
I	2 (28.6)	33 (61.1)	
II	0	1 (1.9)	
III	4 (57.1)	20 (37.0)	
Postoperative adverse event (grade 3 or higher), *n* (%)	2 (28.6)	7 (13.0)	0.273
Postoperative mortality (within 30 days after surgery)	0	0	1.000

Cis, cisplatin; Ipi: ipilimumab; Nivo, nivolumab; P/D: pleurectomy/decortication; PEM: pmemetrexed; MCR, macroscopic complete resection

### Indication of salvage surgery 

**Figure 1:**
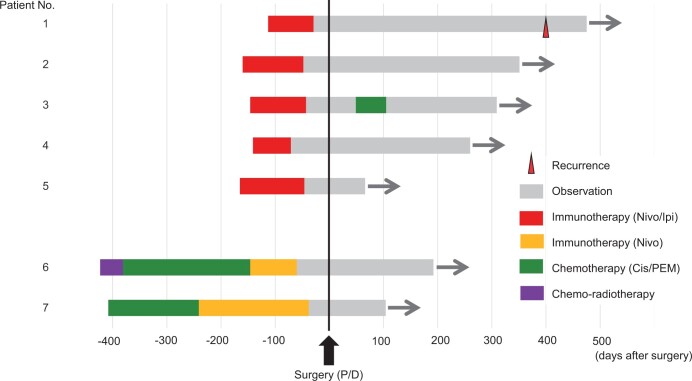
Patient survival data with the treatment modality.

Five patients (patients 1, 2, 3, 4 and 7) received salvage P/D due to conversion to resectability by re-assessment after immunotherapy. Due to significant radiographic response [partial response (PR)], 4 patients (patients 1, 2, 4 and 7) were diagnosed with resectable disease after immunotherapy. One patient (patient 3) had been diagnosed at a previous hospital with unresectable disease due to wide spread invasion to the lung parenchyma. Although immunotherapy had provided no radiographic response, we judged that MCR could be achieved with P/D after careful re-assessment at our institute (Fig. 1 and Supplementary Material, Table S1).

Two patients (patients 5 and 6) received salvage P/D for local relapse after immunotherapy. In addition, salvage surgery was indicated as immunotherapy could not be continued due to immune-related adverse events in 2 patients (patients 4 and 5).

### Feasibility of salvage pleurectomy/decortication

In all 7 patients, MCR was achieved with salvage P/D through thoracotomy. In 1 patient (patient 6), posterior laminectomy was also performed to remove tumour invading into the inter-vertebral foramen (Supplementary Material, Table 1, and Supplementary Material, Table S1).

The operation time was significantly longer in salvage P/D patients than that in non-salvage P/D patients (median, 507 vs 375 min; *P* = 0.030). The intraoperative blood loss might be higher in salvage P/D patients (median, 2573 ml). Grade 3 or higher adverse events were documented after salvage P/D in 2 patients (pneumonitis in patient 7 and persistent air leak with subsequent empyema in patient 6); the incidence might be higher in salvage P/D patients (28.6%) (Supplementary Material, Table S2). No patient died within 30 days after salvage P/D or non-salvage P/D.

### Pathologic findings

The percentage of viable tumour cells in resected specimens was 50% or less in 4 patients who received salvage P/D following immunotherapy (0% in patient 4, <1% in patient 1, 20% in patient 7, 50% in patient 2); in all 4 patients who achieved such pathologic response, significant radiographic response was also documented. In 3 patients with no radiographic response (patients 3, 5 and 6), the percentage of viable cells remained over 50% (Supplementary Material, Fig. S1 and Supplementary Material, Table S1).

### Postoperative prognosis of salvage pleurectomy/decortication

Only 1 patient (patient 3) received postoperative adjuvant treatment (cisplatin plus pemetrexed). With the median follow-up of 271 days, all 7 patients who received salvage P/D were alive. Only 1 patient (patient 1) developed local tumour recurrence, and the other patients were alive without tumour recurrence (Supplementary Material, Table S1 and Fig. 1).

## DISCUSSION

The present study showed that salvage P/D following immunotherapy is a feasible treatment option in highly selected patients with initially unresectable MPM. First, MCR was achieved with P/D in all patients. Second, significant pathologic response (viable cells, ≤ 50%) was achieved in all 4 patients who had radiographic response to immunotherapy. Third, all patients were alive mostly without tumour recurrence. Considering the median recurrence-free survival achieved with trimodality treatment including EPP (10.1 months [9]) and that achieved with P/D (<1 year [10]) for resectable MPM, the prognosis documented in the present study for initially unresectable MPM may be acceptable.

There are several limitations in this study. First, only 7 patients in a single institution were retrospectively analysed. Statistical comparisons of the salvage P/D patients and non-salvage P/D patients may create important biases, as the 2 groups of patients were very heterogenous. To validate the feasibility, a multi-institutional prospective study should be conducted in the future. Second, surgery was associated with longer operation time, higher blood loss and higher ≥ grade 3 morbidity, partly due to difficulty in extrapleural dissection of the parietal pleura probably caused by severe adhesion following immunotherapy. Careful patient selection is essential in considering indication for salvage P/D. Finally, whether salvage P/D provides a significant survival benefit remains unclear. Immunotherapy alone may provide a long-term survival benefit for advanced MPM patients [2, 3]. The present study showed only preliminary results indicating the feasibility of salvage P/D. Long-term follow-up is essential to reveal clinical benefit achieved with salvage P/D following immunotherapy.

To conclude, the present study may indicate the feasibility of salvage P/D following immunotherapy for initially unresectable MPM, which should be validated in future multi-institutional studies.

## Supplementary Material

ivad173_Supplementary_Data

## Data Availability

All relevant data are within the manuscript and its Supporting Information files.
