# Supplementary material for: Salvage pleurectomy/decortication following immunotherapy for malignant pleural mesothelioma
Source: Interdiscip Cardiovasc Thorac Surg. 2023 Nov 15;38(2):ivad173. doi: 10.1093/icvts/ivad173 (PMC10859178; doi:10.1093/icvts/ivad173)
Supplement: ivad173_Supplementary_Data [file ivad173_supplementary_data.zip › SupplementaryFigure1.pdf]

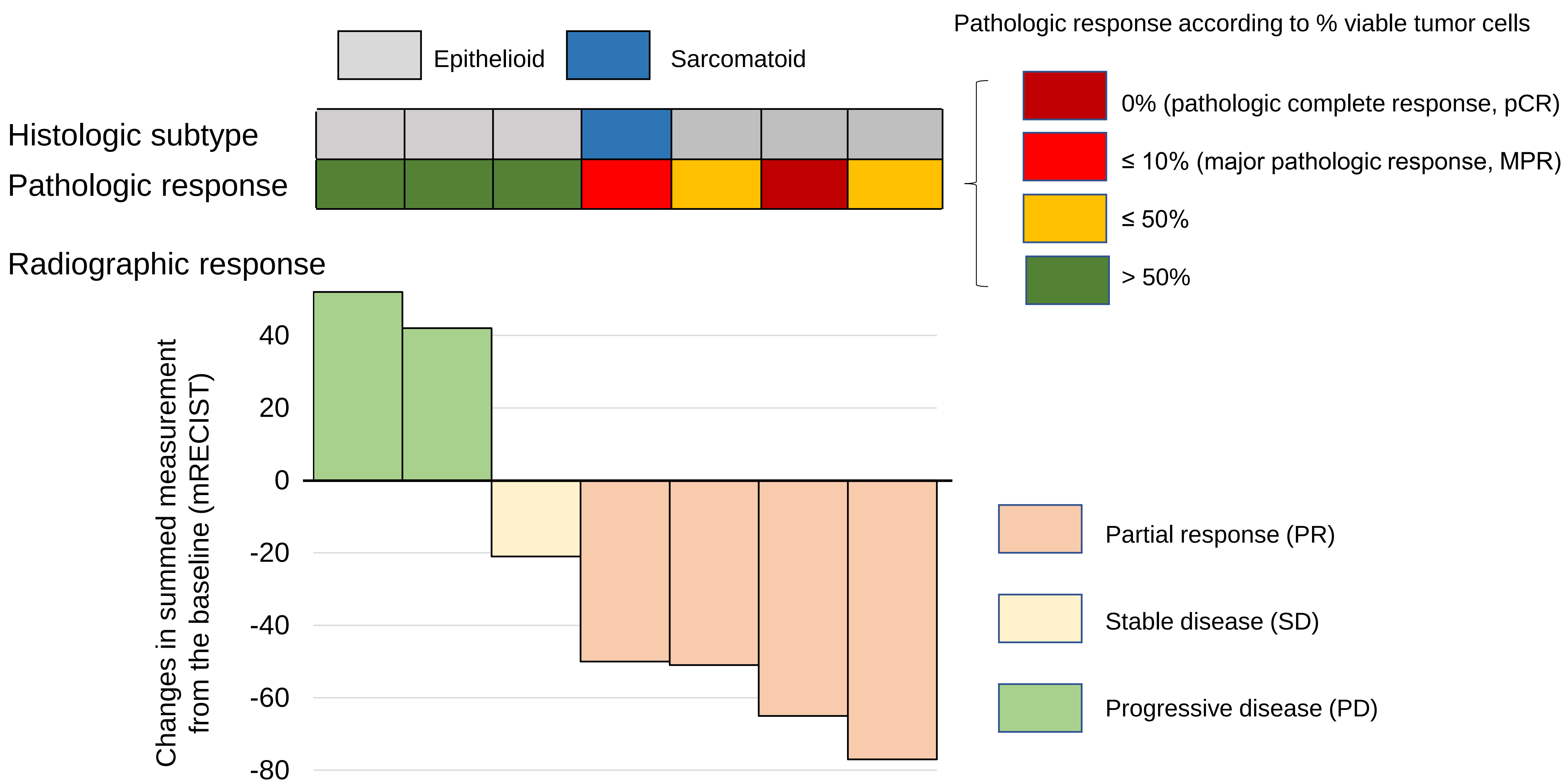

Epithelioid
Sarcomatoid
Pathologic response according to % viable tumor cells
0% (pathologic complete response, pCR)
≤ 10% (major pathologic response, MPR)
≤ 50%
> 50%
Histologic subtype
Pathologic response
Radiographic response
Changes in summed measurement from the baseline (mRECIST)
40
20
0
-20
-40
-60
-80
Partial response (PR)
Stable disease (SD)
Progressive disease (PD)
